# IL-2-free tumor-infiltrating lymphocyte therapy with PD-1 blockade demonstrates potent efficacy in advanced gynecologic cancer

**DOI:** 10.1186/s12916-024-03420-0

**Published:** 2024-05-20

**Authors:** Jing Guo, Chunyan Wang, Ning Luo, Yuliang Wu, Wei Huang, Jihui Zhu, Weihui Shi, Jinye Ding, Yao Ge, Chunhong Liu, Zhen Lu, Robert C. Bast, Guihai Ai, Weihong Yang, Rui Wang, Caixia Li, Rong Chen, Shupeng Liu, Huajun Jin, Binghui Zhao, Zhongping Cheng

**Affiliations:** 1 Department of Obstetrics and Gynecology, Shanghai Tenth People’s Hospital, Tongji University School of Medicine, Shanghai, China; 2 Tongji University School of Medicine, Shanghai, China; 3 Department of Experimental Therapeutics, The University of Texas MD Anderson Cancer Center, Houston, TX USA; 4 Department of Military Health Statistics, Naval Medical University, Shanghai, China; 5 Gynecologic Minimally Invasive Surgery Research Center, Tongji University School of Medicine, Shanghai, China; 6 Shanghai Juncell Therapeutics, Shanghai, China; 7 Department of Radiology, Shanghai Tenth People’s Hospital, Tongji University School of Medicine, Shanghai, China

**Keywords:** Gynecologic malignancies, Tumor-infiltrating lymphocyte, Interleukin-2, Lymphodepletion

## Abstract

**Background:**

Tumor-infiltrating lymphocyte (TIL) therapy has been restricted by intensive lymphodepletion and high-dose intravenous interleukin-2 (IL-2) administration. To address these limitations, we conducted preclinical and clinical studies to evaluate the safety, antitumor activity, and pharmacokinetics of an innovative modified regimen in patients with advanced gynecologic cancer.

**Methods:**

Patient-derived xenografts (PDX) were established from a local recurrent cervical cancer patient. TILs were expanded ex vivo from minced tumors without feeder cells in the modified TIL therapy regimen. Patients underwent low-dose cyclophosphamide lymphodepletion followed by TIL infusion without intravenous IL-2. The primary endpoint was safety; the secondary endpoints included objective response rate, duration of response, and T cell persistence.

**Results:**

In matched patient-derived xenografts (PDX) models, homologous TILs efficiently reduced tumor size (*p* < 0.0001) and underwent IL-2 absence in vivo. In the clinical section, all enrolled patients received TIL infusion using a modified TIL therapy regimen successfully with a manageable safety profile. Five (36%, 95% CI 16.3–61.2) out of 14 evaluable patients experienced objective responses, and three complete responses were ongoing at 19.5, 15.4, and 5.2 months, respectively. Responders had longer overall survival (OS) than non-responders (*p* = 0.036). Infused TILs showed continuous proliferation and long-term persistence in all patients and showed greater proliferation in responders which was indicated by the Morisita overlap index (MOI) of TCR clonotypes between infused TILs and peripheral T cells on day 14 (*p* = 0.004) and day 30 (*p* = 0.004). Higher alteration of the CD8^+^/CD4^+^ ratio on day 14 indicated a longer OS (*p* = 0.010).

**Conclusions:**

Our modified TIL therapy regimen demonstrated manageable safety, and TILs could survive and proliferate without IL-2 intravenous administration, showing potent efficacy in patients with advanced gynecologic cancer.

**Trial registration:**

NCT04766320, Jan 04, 2021.

**Supplementary Information:**

The online version contains supplementary material available at 10.1186/s12916-024-03420-0.

## Background

Cervical, endometrial, and ovarian cancers represent the fourth, sixth, and eighth most commonly diagnosed malignancies among women globally [1]. While initial treatment with surgery and chemotherapy often leads to a high response rate, many patients experience relapse.

Tumor-infiltrating lymphocytes (TILs) have shown great promise in solid tumors [2–5], but were limited in gynecologic cancer [6–8], and conventional TIL therapy involves high-intensity lymphodepleting preconditioning with chemotherapy along with high-dose IL-2 administration [6, 9], which can cause significant toxicity and is only suitable for patients with good performance [6, 10] who receive treatment in specialized clinics [11].

To expand the scope of TIL therapy, a modified regimen was explored, involving a low dose of IL-2 and a mix of cytokines for ex vivo expansion, and the use of a modest lymphodepleting regimen without IL-2 during the infusion phase. In a previous case report, we described the durable complete response of a patient of recurrent cervical cancer with bladder metastasis, who received autologous TIL infusion following the modified regimen with few adverse effects [7].

In this article, we present our pre-clinical results using patient-derived xenografts to investigate the potential of TIL therapy in cervical cancer. We also describe the results of a prospective pilot study of the modified TIL therapy regimen, which demonstrated the safety and feasibility of the approach using low-dose cyclophosphamide lymphodepletion without intravenous IL-2 administration in advanced gynecologic cancer patients. This study is the first clinical trial of TILs generated by a modified regimen in patients with advanced gynecologic cancer.

## Methods

### Design and participants

Our study employed a comprehensive and multi-tiered approach, beginning with the validation of our clinical method through animal experiments. Following the successful validation in animal experiments, we expanded a phase 1, open-label, single-arm trial in patients with advanced gynecologic tumors. Participants were enrolled in clinical trials and received treatments according to a pre-established research protocol. The study adhered to the Declaration of Helsinki and Good Clinical Practice guidelines, and the protocol was approved by the Ethics Committee of Shanghai Tenth People’s Hospital. All patients provided written informed consent before enrollment. Eligibility criteria included being aged 18 to 75 years; having an Eastern Cooperative Oncology Group (ECOG) score of 0 or 2; estimated life expectancy > 3 months; having histologically confirmed and standard treatment failed or standard treatment-intolerant stage IIIC or IV gynecologic cancer; having one or more lesions (minimum 15 mm in diameter) that could be surgically removed for TIL generation; having residual measurable disease after resection as defined by Response Evaluation Criteria in Solid Tumors (RECIST), version 1.1 [12]; being recruited between Jan 24, 2021, and Dec 4, 2022 at Shanghai Tenth People’s Hospital. A full overview of eligibility criteria is provided in the Supplement protocol.

The scheme of the trial is shown in Additional file 1: Fig. S1. Fludarabine and IL-2 were not used in our trial. Patients received three consecutive daily infusions of cyclophosphamide (20 mg/kg/day) from day−5 to day−3, and oral administration of hydroxychloroquine (600 mg once) on day−5. On day 0, within 50 to 70 min following the administration of anti PD-1 antibody (100 mg, sintilimab, Innovent) patients received a single intravenous adoptive transfer of 1×10^9^ to 3×10^10^ TILs in 30 to 120 min.

### TIL Manufacturing

The identical TIL generation protocol used in both the pre-clinical and clinical sections of this study was the same as in a previous study [7]. Resected tumor was processed and shipped to a Good Manufacturing Practice (GMP) facility of Shanghai Juncell Therapeutics. The tumor fragments were subjected to culture in a pre-Rapid Expansion Protocol (pre-REP) medium containing X-VIVO 15 plus rhIL-2 (201-GMP-01 M, R&D) (2000 IU/mL), rhIL-7 (207-GMP-01 M, R&D) (10 ng/mL) and rhIL-15 (247-GMP-01 M, R&D) (10 ng/mL). After the pre-REP culture, TILs were incubated for 2 days in a plate precoated with anti-CD3 antibody (317,302, Biolegend) and anti-CD28 (MAB342-500, R&D) aiming to be reactivated before they were transferred to G-REX100 harboring a REP culture medium containing X-VIVO 15 plus rhIL-2 (300 IU/mL) for a further linear expansion. The final TIL product for autologous adoptive transfer was prepared as a cryopreserved drug product (CDP) containing 1×10^10^ cells aliquoted in 100 mL. Before it was released, we characterized cell subtypes of the TIL product by staining cell surface markers on TILs through flow cytometry aiming to detect invigorated T cells.

### Characterization of the infusion product

For cell preparation for flow cytometry, cells were harvested, then resuspended in a stain buffer (Biolegend, 420201), and incubated with titrated fluorochrome-conjugated antibodies for about 30 min in the refrigerator and dark. Cells were then washed with stain buffer and resuspended with stain buffer followed by data acquisition with full spectrum flow cytometry. Flow cytometry was used to evaluate transduction efficiency and expression of select cell surface markers. The following anti-human flow cytometry antibodies were used in this report: CD3 (317340), CD4 (344618), CD8a (301038), CD25 (356,104), CD28 (302950), CD39 (328236), CD44 (338,808), CD45 (304036), CD56 (362542), CD57 (359608), CD69 (310965), CD137 (309808), CD150 (306306), CCR2 (357210), CCR5 (359108), CCR7 (353254), CX3CR1 (341626), CXCR1 (320610), CXCR2 (320722), CXCR3 (353728), LAG-3 (369350), PD-1 (329950), TIGIT (372714), and TIM-3 (345034); all the antibodies above are from Biolegend. APC Streptavidin (554,067) and anti-CD137 body (550,890) are from BD. Flow cytometry data were analyzed using FCS express 7. Analysis of the cell population was performed based on four-step criteria. The first step was the gating of lymphocytes on the basis of forward and side scatter characteristics. The second step was the removal of dead cells, debris, and doublets. The third step was the gating of T cells and non-T cells on the basis of CD3 expression. From the CD3-gated cells, CD4 versus CD8-positive cells were plotted which will allow the identification of discrete CD4 and CD8 cell populations. Subsequent analyses were done on these individual cell populations.

### Patient-derived xenografts model

Patient-derived xenografts (PDX) were established from a local recurrent cervical cancer patient. Female B-NDG mice were purchased from Biocytogen and maintained under specific pathogen-free conditions. Patient-derived xenografts (PDX) were established from a local cervical cancer recurrence patient. The tumor tissue was implanted under isoflurane anesthesia in the RAS-4 rodent anesthesia system (PerkinElmer). After tumor implantation, 16 mice with xenografts reaching ~ 30 mm^3^ in volume were randomly divided into two groups: Group control, which received cryoprotectant, and Group TIL, which received homologous TILs generated from the same patient. On day 0, group control received 0.2 mL cryoprotectant, and group TIL received 1.64×10^7^ TILs suspended in 0.2 ml cryoprotectant. No intravenous IL-2 was administrated throughout the experiment. Tumor volume was measured 3 times every week, using vernier calipers and calculated using formula *A*×*B*^2× ^0.5, where *A* and *B* represented the longest and shortest diameters of the tumor, respectively. After 40 days, the mice were euthanized using CO_2_. Peripheral blood and solid tumors were collected for flow cytometry and multiplexed immunohistochemistry (mIHC) analysis.

The peripheral blood of the mouse was analyzed by flow cytometry, and data were analyzed using flow cytometry data were analyzed using FCS express 7. Flow cytometry was used to evaluate transduction efficiency and expression of select cell surface markers. The following anti-human flow cytometry antibodies were used in this report: CD3 (317340), CD4 (344618), CD8a (301038), CD39 (328236), CD45 (304036), CD57 (359608), CD62L (304,814), CCR7 (353,218), LAG-3 (369,350), and PD-1 (329950); all the antibodies above are from Biolegend. Flow cytometry data were analyzed using FCS express 7.

Multiplexed immunohistochemistry (mIHC) was performed by staining 4-um-thick formalin-fixed, paraffin-embedded whole tissue sections with standard, primary antibodies sequentially and paired with TSA 6-color kit (M-D110061-50 T, Yuanxibio). Then by staining with SN470. For example, starting from the second to fifth rounds of staining, slides were washed in TBST buffer and then transferred to preheated EDTA solution (100 ℃ mIHC) before being heat-treated for 15 min. Slides were cooled in the same solution to room temperature, incubated with anti-CD4 (ab133616, abcam) for 60 min, and then treated with peroxidase-conjugated (HRP) secondary antibody (DS9800, Leica) for 10 min. Then labeling was developed for a strictly observed 10 min, using TSA 620 per the manufacturer’s direction. Between all steps, the slides were washed with Tris buffer. The same process was repeated for the following antibodies/fluorescent dyes, in order: anti-CD8 (BX50036, Biolynx)/TSA 520, anti-Ki-67 (BX50040, Biolynx)/TSA 570, anti-CD56 (3576, CST)/TSA 670, and anti-pan CK (GM351507, Gene Tech, SA 440). Each slide was then treated with 2 drops of SN470 (A11010-100 T; Yuanxibio), washed in distilled water, and manually covered slipped. Slides were air dried and took pictures with the Pannoramic MIDI II tissue imaging system (3DHISTECH). Images were analyzed using Indica Halo software.

### Treatment evaluation

The primary endpoint was safety. Safety assessments were conducted for all patients at baseline and regular intervals. The severity of all adverse events was graded based on Common Terminology Criteria for Adverse Events (CTCAE), version 5.0. The secondary endpoints were objective response rate (ORR), disease control rate (DCR), duration of response (DOR), and progression-free survival (PFS). Antitumor activity was assessed by CT scanning or MRI at 4 to 6 weeks after TIL infusion, then every 4–6 weeks for 1 year, and then every 6 months thereafter for up to 3 years based on Response Evaluation Criteria in Solid Tumors version 1.1. The response evaluation is detailed in the Supplementary protocol. Peripheral blood samples were collected for blood hematology analysis and serum biochemistry analysis. Additional post hoc exploratory analyses assessed potential associations between profiles of peripheral TILs with clinical outcomes.

###  Cell receptor sequencing and bioinformatic processing

Total RNA was extracted using the miRNeasy Mini Kit (Catalog 217,084). RNA was used to build libraries with the QIAseq Immune Repertoire T-cell Receptor Panel (Catalog 333,705-IMHS-001Z). With this kit, RNA is reverse transcribed with a pool of gene-specific primers against the C (constant) region for the T cell receptor alpha, beta, gamma, and delta genes. The reverse transcribed cDNA is then used in a 5′ ligation reaction which adds an oligo that contains one side of the sample index and a unique molecular index. Following reaction cleanup, a single primer extension is used to capture the T cell receptor using a pool of gene-specific primers. The resulting captured sequences are amplified and purified using QIAseq beads. The libraries then are sample indexed on the other side by using a unique sample index primer and a universal primer. The final dual sample indexed PCR fragment is then quantitated for abundance using the Agilent™ 2100 Bioanalyzer™. Illumina libraries were sequenced (150-bp paired-end reads) using the Illumina NovaSeq6000 system. Raw sequence reads were processed using MIXCR [13] version 4.0.0. VDJtools [14] was applied to MIXCR output to derive clonotype tracking using the command TrackClonotypes. One million normalized reads were randomly selected for further analysis. The MOI for determining the similarity between samples ranges from 0 and 1, representing minimal and maximal similarity, respectively.

### Statistical analysis

No sample size calculations were performed. Safety was evaluated in all patients receiving at least one dose of chemotherapy. Continuous variables were reported as mean with standard deviation (SD) or median with range. Categorical data are presented as numbers and percentages where applicable. Responses after treatment were reported with associated 95% confidence intervals. Overall survival, PFS, ORR, DCR, and DoR were calculated using the Kaplan–Meier method, and 95% confidence interval was included for medians and curves. GraphPad Prism 8.0.2 (GraphPad Software) was used for graphing and statistical analysis. MOI was analyzed using R package immunarch [15].

## Results

### Pre-clinical section

**Fig. 1:**
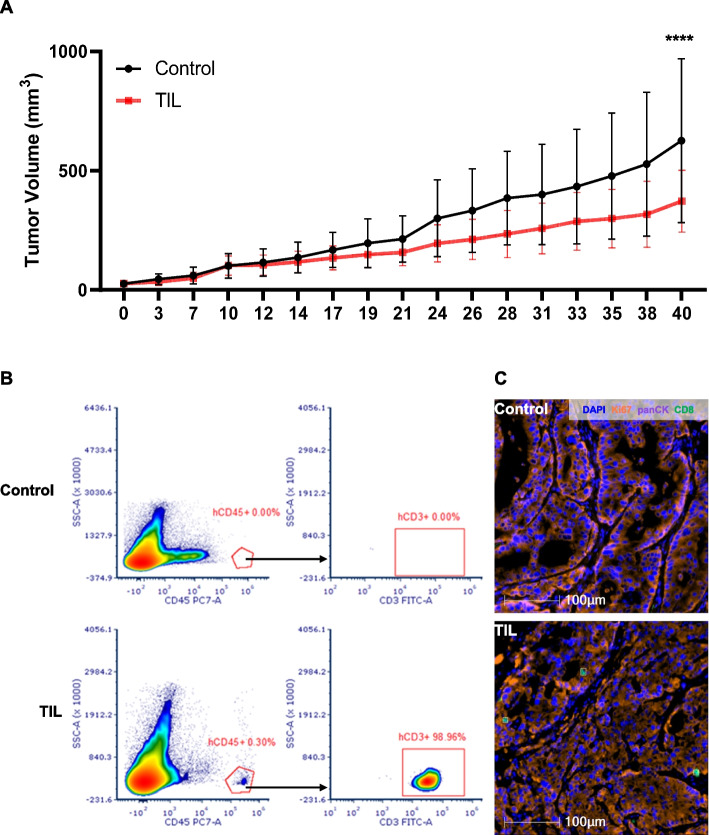
Antitumor activity and homing ability of TILs in PDX model. **A** PDX models were established in B-NDG mice from a local cervical cancer recurrence patient which were randomly divided into two groups: group control, which received cryoprotectant, and group TIL, which received homologous TILs generated from the same patient. Tumor volume was measured three times per week, using vernier calipers and calculated using formula *A*×*B*^2× ^0.5, where *A* and *B* represented the longest and shortest diameters of the tumor, respectively. Tumor volumes in group TIL were smaller than in group control (*p* < 0.0001). **B** The peripheral blood of PDX mice was obtained on day 40 post infusion for flow cytometry. **C** The tumor samples of PDX mice were obtained on day 40 post infusion for multiplexed immunohistochemistry (mIHC), using the following markers: Ki67, panCK, CD8, and DAPI. The scale bar is 100 µm. PDX, patient-derived xenografts

TILs were successfully derived and generated from a local recurrent cervical cancer patient. The characteristics of TIL were accessed by flow cytometry (Additional file 1: Fig. S2). A high proportion of T cells was detected in generated cells, which showed a high level of CD8 and a relatively low level of exhaustion markers (CD57 15%, LAG-3 33%, and PD-1 0.4%). As shown in Fig. 1A, the matched PDX model showed a smaller tumor volume in the group TIL than the group control (*p* < 0.0001). At the 40-day post-TIL infusion, a CD45^+^ T cell count of 0.3% was detected in the mouse’s peripheral blood (Fig. 1B), with nearly all the CD45^+^ T cells being CD3 positive (98.96%). Multiple immunofluorescent staining revealed co-localization of CD8^+^ cells and pan-CK positive cells (Fig. 1C).

### Immunophenotypic characteristics of TIL

We performed flow cytometry on TIL products to determine the immunophenotypic characteristics of the infused T cells. As shown in Additional file 1: Fig. S3, the majority of infused TILs were CD45^+^CD3^+^ T lymphocytes (range, 74.6–99.3%). After ex vivo expansion, we found that the proportion of CD45RA^ − ^CCR7^ − ^effector memory T cells (TEM) were high (range, 57.8–99.0%), with a small proportion of CD45RA^ − ^CCR7^+^ central memory T cells (TCM). The activation-associated cell surface markers, such as CD25, CD28, CD39, CD69, CD44, and CD150 were highly expressed, while the exhaustion-associated markers, including PD-1, LAG3, TIGIT, TIM3, and CD57 were low or modestly elevated. In addition, several chemokine receptors, which play a role in cell trafficking (e.g., CCR2, CCR5, and CXCR3), were highly expressed, and CX3CR1 expression varied among patients.

### Patient characteristics

A total of 16 patients with advanced gynecological cancers were enrolled and received TIL infusion. Most patients were discharged within 7 days or less after the lesion excision for TIL culture. We successfully expanded TILs from all enrolled patients (100%), demonstrating that the protocol of expanding TILs from patients with a recurrent gynecologic cancer was feasible. Two patients (ID 09, 11) lost follow-up due to the coronavirus disease 2019 (COVID-19) pandemic.

Among the 14 evaluable patients, 6 were diagnosed with ovarian cancer, 5 with cervical cancer, 2 with endometrium cancer, and 1 patient with peritoneal cancer. The median age of patients was 51.5 years (range 33–65). Patients had a mean of 3.4 lesions, most of which were bulky with a mean sum of target lesion diameters of 43.6 mm. Patients had a median ECOG performance status score of 2 (range 1–3). The patients had received a mean of 3.4 lines of systemic therapy, and 6 patients had received anti-PD-1 or CTLA4 antibody therapy before TIL treatment (Additional file 2: Table S1). TILs were successfully expanded and infused for all 14 evaluable patients to a median dose of 20.9 billion lymphocytes (range 12.5–50.5), with a median of 71% CD8^+^ of CD3^+^ T lymphocytes (range 5–97%) (Additional file 1: Table S1).

### Safety and adverse events

AEs included lymphopenia (93%), leukopenia (79%), and neutropenia (71%) (Table 1) with the most common grade 3 or 4 AEs, which were primarily attributed to lymphodepletion. Other common nonhematologic adverse events included fever, nausea, and diarrhea, which were attributed to the DMSO cryoprotectant (media the T cells stored). No treatment-related mortality occurred. Following lymphodepletion, patients recovered lymphoid and myeloid lineages (Additional file 1: Fig. S4A, 4D, 4G, and 4 J), with neutrophil counts recovering within a median of 10 days (range 7–14). Stable levels were observed in both the hemoglobin level and platelet count (Additional file 1: Fig. S4B, 4E, and 4H). Basal levels of cytokines including IL-2, IL-4, IL-6, IL-8, IL-10, TNF-alpha, and IFN-gamma showed a transit elevation followed by stable status in peripheral blood, especially the level of IL-6, which is the central mediator of toxicity associated with cytokine release syndrome (CRS) (Additional file 1: Fig. S4C, 4F, 4I, 4 K, and 4L). Most treatment-emergent adverse events had resolved within 1 month after TIL treatment.

**Table 1:** Adverse events

Adverse event	Grade 1	Grade 2	Grade 3	Grade 4
Leukopenia	0 (0%)	2 (14.3%)	5 (35.7%)	6 (42.9%)
Lymphopenia	0 (0%)	1 (7.1%)	8 (57.1%)	5 (35.7%)
Neutropenia	1 (7.1%)	2 (14.3%)	4 (28.6%)	6 (42.9%)
Monocytopenia	3 (21.4%)	0 (0%)	0 (0%)	0 (0%)
Thrombocytopenia	1 (7.1%)	3 (21.4%)	0 (0%)	0 (0%)
Anemia	3 (21.4%)	6 (42.9%)	0 (0%)	0 (0%)
Fever	5 (35.7%)	9 (64.3%)	0 (0%)	0 (0%)
Chills	2 (14.3%)	0 (0%)	1 (7.1%)	0 (0%)
Urethritis	1 (7.1%)	0 (0%)	0 (0%)	0 (0%)
Nausea	2 (14.3%)	0 (0%)	0 (0%)	0 (0%)
Emesis	5 (35.7%)	0 (0%)	0 (0%)	0 (0%)
Diarrhea	1 (7.1%)	0 (0%)	0 (0%)	0 (0%)
Abdominal distension	0 (0%)	1 (7.1%)	0 (0%)	0 (0%)
Constipation	1 (7.1%)	0 (0%)	0 (0%)	0 (0%)
ALT increased	1 (7.1%)	0 (0%)	0 (0%)	0 (0%)
AST increased	2 (14.3%)	0 (0%)	0 (0%)	0 (0%)
Creatinine increased	1 (7.1%)	0 (0%)	0 (0%)	0 (0%)
Hyperuricaemia	3 (21.4%)	0 (0%)	0 (0%)	0 (0%)
Fatigue	0 (0%)	1 (7.1%)	0 (0%)	0 (0%)
Mucositis oral	1 (7.1%)	0 (0%)	0 (0%)	0 (0%)
Rash	2 (14.3%)	0 (0%)	0 (0%)	0 (0%)
Pruritus	1 (7.1%)	0 (0%)	0 (0%)	0 (0%)
Skin Sensitisation	1 (7.1%)	0 (0%)	0 (0%)	0 (0%)
Pachulosis	1 (7.1%)	0 (0%)	0 (0%)	0 (0%)
Sinus tachycardia	1 (7.1%)	0 (0%)	0 (0%)	0 (0%)
Alopecia	1 (7.1%)	0 (0%)	0 (0%)	0 (0%)

### Clinical activity

**Fig. 2:**
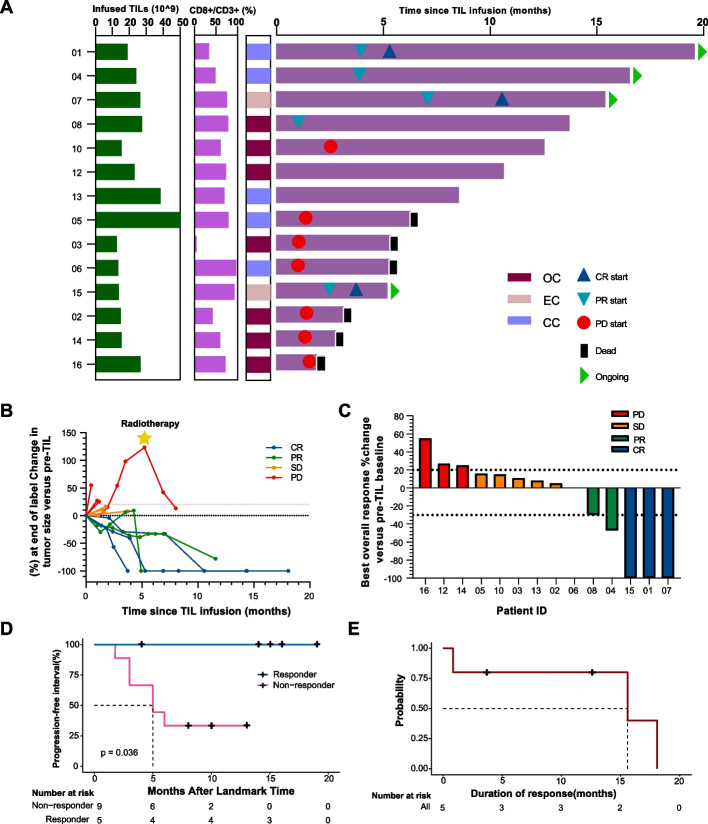
Clinical responses of modified TIL therapy. **A** Swimmer plot of treatment duration. The length of each bar represents the treatment duration for each patient. Three complete responses were ongoing 19.5, 15.4, and 5.2 months. Patient 04 had a partial response, with one lesion remaining in complete remission for 17 months, while another small non-target lesion progressed 1 month after TIL infusion, which was removed by surgery 7 months after infusion maintaining clinical remission up to the present. **B** Spider plot of measurements of target lesions at each timepoint. **C** Waterfall plot of the best change in the sum of target lesion size compared with that at baseline. The horizontal line at 20% indicates progressive disease, and at − 30% indicates the threshold for defining an objective response in the absence of non-target disease progression or new lesions according to RECIST 1.1. **D** Kaplan–Meier curves for overall survival in responders and non-responders. CR, complete response; PD, progressive disease; PR, partial response; SD, stable disease. **E** Kaplan–Meier curve demonstrates the duration of response, defined as the time from the achievement of a response to progression. Among 5 responders, the median duration of the response was 15.6 months (95% exact lower confidence limit =15.6 months; upper confidence limit has not been reached)

Among 14 evaluable patients, the investigator-assessed ORR was 36% (5/14, 95%CI 16.3–61.2) and the DCR was 71% (10/14, 95%CI 45.4–88.3), with 3 (21%) complete responses (CRs), 2 (14%) partial responses (PRs), and 5 (36%) patients showing stable disease (SD) (Fig. 2A, B, and C; Additional file 2: Table S2). Three complete responses were ongoing 19.5, 15.4, and 5.2 months (Fig. 2A).

Among 14 evaluable patients, the median overall survival of responders was not reached after TIL treatment, representing a significant improvement in the survival time of advanced gynecologic cancer patients compared to non-responders (Fig. 2D). Among 5 responders, the median duration of the response was 15.6 months (95% exact lower confidence limit = 15.6 months; upper confidence limit has not been reached) (Fig. 2E). Three complete responses were ongoing 19.5, 15.4, and 5.2 months, respectively. Patient 04 had a partial response, with one lesion remaining in complete remission for 17 months, while another small non-target lesion progressed 1 month after TIL infusion, which was removed by surgery 7 months after infusion maintaining clinical remission up to the present.

### Persistence and subtypes of T cells

**Fig. 3:**
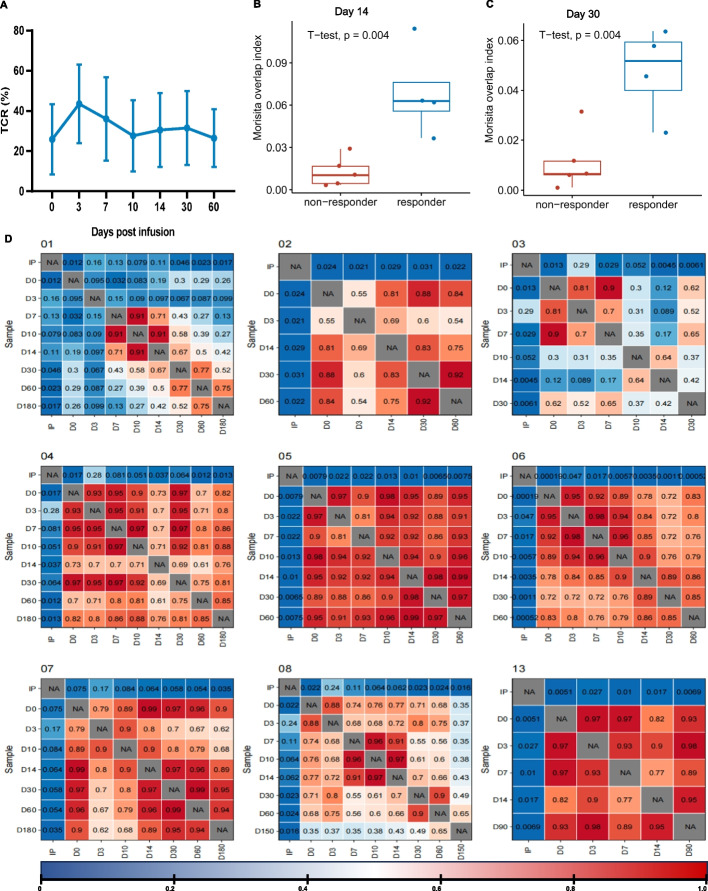
The association between clinical response with dynamics of TCR. **A** The percentages of infused TCR clonotype in peripheral blood at different time points post TIL infusion. **B** and **C** Differences of MOI between responders and non-responders on day 14 and day 30 respectively. **D** Dynamic changes of MOI post-infusion per patient. MOI, Morisita overlap index

TCRβ sequencing showed the infused TIL clonotypes largely replaced the patients’ baseline T cell repertoire, and then gradually decayed in proportion in the ensuing follow-up, indicating clonally expanded cells may infiltrate to tumor tissue (Fig. 3A).

Morisita overlap index (MOI) was utilized to quantify the similarity of TCR clonotype between infused TILs and circulating T cells. We found that the MOI of responders was higher than that of non-responders on Day 14 (*p* = 0.004) and Day 30 (*p* = 0.004), respectively (Fig. 3B and C).

### Alterationof CD8 + /CD4 + relative to infused TIL products

**Fig. 4:**
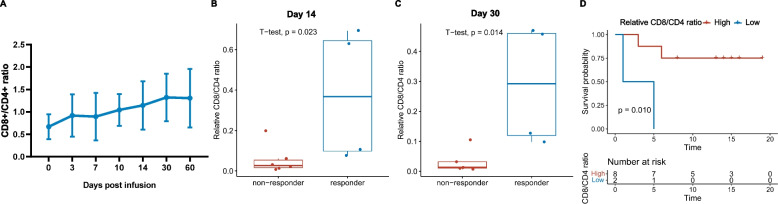
The association between clinical response with dynamics of CD8^+^/CD4^+^ ratio. **A** Circulating CD8^+^/CD4^+^ ratio at different time points post TIL infusion. **B** and **C** Differences of relative CD8^+^/CD4^+^ ratio (comparing with infused TIL products) in responders and non-responders on day 14 and day 30, respectively. **D** Kaplan–Meier curves for overall survival in patients with high or low relative CD8^+^/CD4^+^ ratio

The ratio of circulating CD8^+^/CD4^+^ was raised after TIL infusion (Fig. 4A). Responders had a higher relative CD8^+^/CD4^+^ ratio (compared with infused TIL-product) than non-responders on day 14 and day 30 (*p* = 0.023, *p* = 0.014, Fig. 4B and C). The ORR and DCR for the high relative CD8^+^/CD4^+^ ratio group were 50% and 87.5%, respectively, and the ORR and DCR for the low relative CD8^+^/CD4^+^ ratio group were 0 and 50.0%, respectively (Additional file 1: Fig. S5). A higher relative CD8^+^/CD4^+^ ratio on Day 14 indicates a longer survival (*p* = 0.010, Fig. 4D).

### Representative patients’ analysis

**Fig. 5:**
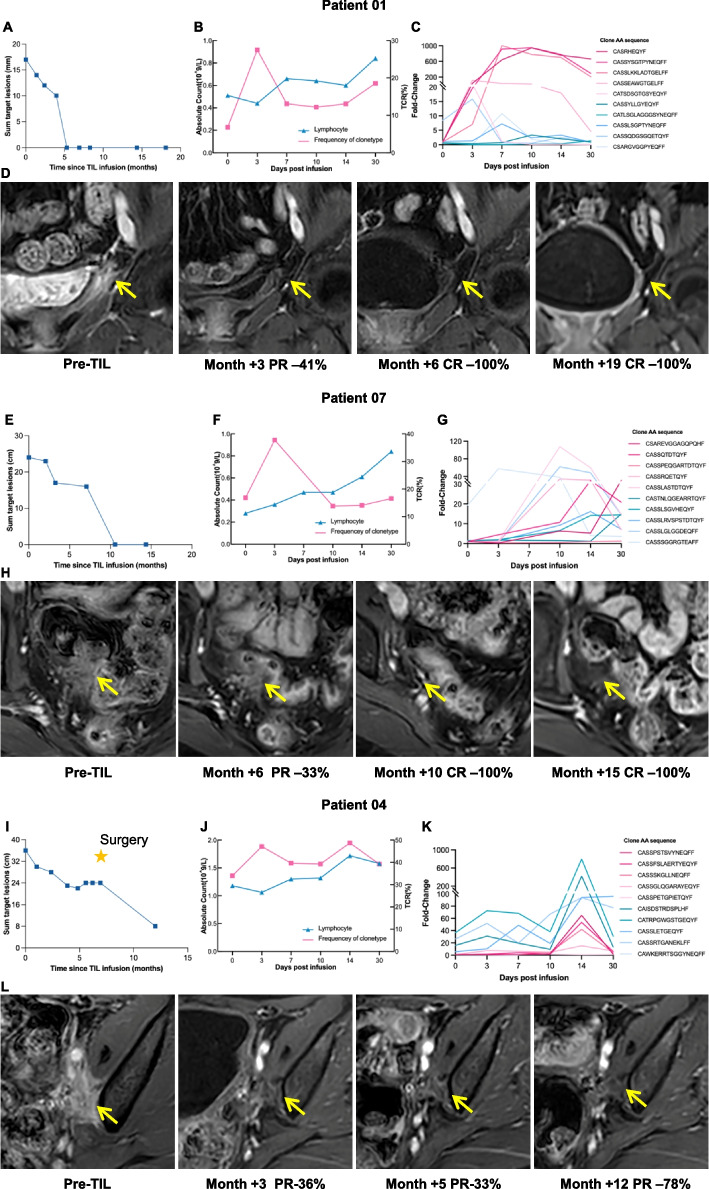
MRI images and TCR clonotypes in the representative patients. Patient ID 01 had progressive metastases of cervical cancer in the bladder followed by a complete response to TILs. **A** The sum of radiographic target lesions is shown over time with representative contrast-enhanced axial MRI images in patient ID 01. **B** Changes in T cell clonotypes and lymphocytes in peripheral blood after infusion. **C** Changes in exogenous and endogenous TCR clonotypes within 30 days after TIL infusion. **D** MRI images of target lesions during the follow-up. Patient ID 07 had progressive metastases of endometrial cancer in the anterior wall of the rectum followed by a complete response to TILs. **E** The sum of radiographic target lesions. **F** Changes in T cell clonotypes and lymphocytes. **G** Changes in exogenous and endogenous TCR clonotypes within 30 days after TIL infusion. **H** MRI images of target lesions during the follow-up. Patient ID 04 had progressive metastases of cervical cancer in the bladder followed by a partial response to TILs. **I** Sum of radiographic target lesions. **J** Changes in T cell clonotypes and lymphocytes. **K** Changes in exogenous and endogenous TCR clonotypes within 30 days after TIL infusion. **L** MRI images of target lesions during the follow-up

We analyzed the frequency of circulating TIL clones of three patients (ID 01, 07, 04) who achieved sustained clinical responses. We distinguished exogenous TCR clonotypes, which were shared with the TIL infusion product, from endogenous TCR clonotypes, which were not. Both exogenous and endogenous TCR clonotypes increased after TIL infusion. For example, patient ID 01 had recurrent cervical cancer with bladder metastasis and achieved a sustained complete response for over 1.5 years after TIL treatment (Fig. 5A and D). After infusion, circulating T cell clonotypes increased markable, which was consistent with the lymphocytes (Fig. 5B). Exogenous T cell clonotypes and endogenous T cell clonotypes increased, suggesting a putative dominant role in tumor killing (Fig. 5C). Similarly, patient ID 07 had recurrent endometrial cancer with rectal metastasis, her complete response has lasted for 1.5 years after TIL treatment (Fig. 5E and H). In total, 358 T cell clonotypes had more than a tenfold increase, including 240 exogenous T cell clonotypes and 118 endogenous T cell clonotypes (Fig. 5G). Patient ID 04 had a confirmed partial response (Fig. 5I), with 2052 exogenous T cell clonotypes and 123 endogenous T cell clonotypes increasing by tenfold (Fig. 5K and L). These results suggest that some T cell clones from the infused TILs greatly proliferated in vivo and may have activated endogenous tumor-reactive T cells to cooperate in killing tumors.

## Discussion

Clinical application of conventional TIL therapy was limited by the severe toxicity induced by high-intensity lymphodeplete along with high-dose IL-2 administration. Our results demonstrated that the modified regimen was feasible both in the T cell manufacturing and in the clinical phase. We observed a 36% response with a DCR of 71%, which was comparable to conventional TIL therapy with ORR ranging from 35 to 44% in solid tumors [8, 16].

High doses of IL-2 have been approved for conventional adoptive cell therapy (ACT) in cancer treatment for promoting the development and activity of all T cell subsets [17, 18], including regulatory T (Treg) cells which dampen immune responses against tumors. To combat this dichotomous effect, high-intensity lymphodepletion has been used in conventional TIL therapy regimens to ensure that IL-2 stimulates the infused TILs while preventing innate Tregs activation. Also, IL-2 has side effects of vascular leak syndrome [19].

Given the conventional ACT in cancer treatment producing iatrogenic severe disease, it is rational to modify the protocol of TIL therapy. In the TIL generation phase of our modified regimen, reducing the dose of IL-2 in the culture medium is the most important revolution which was the basis for eliminating IL-2 intravenous injection after TIL infusion. All tumor samples, both pre-clinical and clinical, successfully underwent ex vivo lymphocyte expansion using the modified protocol. Particularly, immunodeficient PDX model B-NDG mice were utilized, which means not only exogenous but endogenous IL-2 was eliminated throughout the experiment. Despite the absence of IL-2, TIL displayed in vivo antitumor activity, suggesting TILs can proliferate in vivo without IL-2 (Fig. 1A). Elevated levels of serum IL-7 and IL-15 following lymphodepletion were supposed to promote TIL proliferation [20, 21]. In the clinical section, TCR repertoire sequencing on different time points showed that the TCR clone frequency increased along with time post infusion, and the infused TILs could promote endogenous tumor-reactive T cell activation. The stimulation might have an impact on distinct phases after infusion, as the proliferation of TILs with different TCR clones occurs asynchronously. The kinetics of the TCR repertoire had verified that in vivo proliferation of TILs could omit intravenous IL-2 administration. Without IL-2 promotion to Treg, a low-intensity lymphodepletion regimen may be sufficient for TIL infusion. Meanwhile, the homing ability of TIL to tumor sites was confirmed by the low circulating frequency of TILs on day 40 (Fig. 1B), as well as the co-localization of anti-CD8 and anti-panCK (Fig. 1C). These localized antitumor effects contributed to the low systemic toxicity. Obviously, the most common SAEs, including lymphopenia, leukopenia, and neutropenia, were primarily attributed to lymphodepletion, which were recovered within 1 week post TILs infusion. As expected, negligible adverse effects were observed in patients who did not receive intravenous IL-2 and high-intensity lymphodepletion, which supports our hypothesis that this regimen can be performed in most clinic centers.

Besides potent T cell growth factor activity, IL-2 can augment CD8 T cell activities against PD-L1-expressing target cells by reducing PD-1 expression [22]. During the infusion phase of our treatment, IL-2 removal led to increased safety but resulted in a loss of PD-1 inhibition. Therefore, a low-dose anti-PD-1 antibody was utilized in our regime for its capacity to address the immunosuppressive tumor microenvironment as a replacement of IL-2 [23] rather than its conventional antitumor activity. Furthermore, of the patients in our study, four enrolled in the study after progression from previous exposure to PD-1 antibodies. Despite resistance to anti-PD-1, they showed promising results post-trial (1 CR, 1 PR, and 2 SD). Based on the results of these 4 patients, we are confident in excluding the PD-1 antibody as part of antitumor efficacy in this therapeutic regimen. Treatment with PD-1 blocking antibodies was widely used front-line therapy for patients with solid tumors. Among those patients who initially respond to PD-(L)1 blockade, over half will eventually develop progression. Our finding is particularly encouraging for patients who had failed to ICIs or standard therapy.

In our study, all the responders had sustained clinical benefit (Fig. 2A). T cell responses may vary based on their kinetics which were supposed that associated with the durability of responses. We examined the similarity between the peripheral blood T cells after TIL infusion and the infused TILs by utilizing the MOI. MOI takes into account both the abundance and composition of T cells. On day 14 and day 30 post-infusion, responders and non-responders showed significant differences in MOI, indicating continued proliferation of infused TIL and a positive correlation existed between MOI and clinical response (Fig. 3B and C). A higher MOI between infused TILs and peripheral T cell pool post-infusion suggests a more successful proliferation of infused TIL. And this homeostatic T cell proliferation is a physiologic process by which effective antitumor autoimmunity can be elicited [24]. These findings suggest that MOI could predict the clinical response.

The phenotype of T cells administered to patients often correlates with antitumor reactivity [25]. Since high-dose IL-2-injection induced T cell exhaustion [26], less differentiated and more stem-like TILs were preferred to improve the persistence of TILs in vivo [27]. TCMs, which are less differentiated, have been found to have a crucial role in T cell persistence, compared to more differentiated effector memory T cells (TEMs) [28–30]. However, similar to the previous study, a considerable proportion of TEM was detected in our TILs (Additional file 1: Fig. S3) after mega-expansion in ex vivo. Nevertheless, our study suggests that without high-dose IL-2 intravenous injection, the progression of T cell exhaustion is slowed down, thus contributing to the long persistence of TILs. In addition, proportions of circulating CD8^+^ T cells in the peripheral circulation were an independent prognostic factor in patients with head and neck squamous cell carcinoma[31]. Consistent with the findings of previous studies, our results showed that CD8^+^/CD4^+^ T cell ratio increased continuously post-infusion (Fig. 4A), and higher relative CD8^+^/CD4^+^ T cell ratio contributed to survival benefit in patients with gynecologic cancer (Fig. 4B, C and D). As well known, CD8 CTLs play a critical role in antitumor immunity [32, 33]. Thus the CD8^+^/CD4^+^ ratio on day 14 should be an effective prognosis factor for TIL therapy which is important for clinical decision-making.

This study has some inherent limitations. First, the small sample size and various tumors of patients might result in statistical bias and limit the statistical power of this study to some degree. Second, the COVID-19 pandemic continued throughout the study making it difficult to control research quality, resulting in two patients losing follow-up. Further studies, randomized against standard treatments in other solid tumors, are necessary to validate the clinical benefits of optimized TIL therapy regimens. Several phase 1 studies, such as TIL therapy in patients with advanced malignant solid tumors (NCT05417750), or in patients with advanced breast cancer (NCT05142475), are in progress or planned on the basis of our findings in this study.

## Conclusions

The modified TIL regimen showed manageable safety and encouraging clinical benefits, promising treatment in patients with advanced gynecologic cancer, especially in elderly patients or patients who cannot tolerate high-toxicity treatment. In the early post-infusion stages, MOI between infused TILs and circulating T cells, as well as circulating alterations in CD8^+^/CD4^+^ ratios, proved to have prognostic value for survival.

### Supplementary Information

Additional file 1: Figure S1-S5. Fig. S1-Scheme of clinical study. Fig. S2-Characteristics of TIL used in PDX model. Fig. S3-Characteristics of TIL products. Fig. S4-Analysis of peripheral blood pre- and post-infusion of TILs. Fig. S5-The association between clinical response with dynamics of CD8 + /CD4 + ratio. Supplementary Fig. 1. Scheme of clinical study. Day count was relative to TIL infusion. Preconditioning regimen: cyclophosphamide (20 mg/kg/day) from day -5 to day -3, and oral administration of hydroxychloroquine (600 mg once) on day -5. On day 0, following the administration of anti-PD-1 antibody (100 mg, sintilimab, Innovent) patients received a single intravenous TILs. Supplementary Fig. 2. Characteristics of TIL used in PDX model. Flow cytometry analysis of the TIL generating for PDX model. (A) CD45 positive cells in generated cells. (B) Expression of CD3 in CD45 positive cells. (C) analysis of CD45 and CD3 double positive cells. LAG-3, Lymphocyte activation gene 3; PD-1, programmed death-1; PDX, patient-derived xenografts; TCM, central memory T cells; TEM, effector memory T cells; TIL, Tumor-infiltrating lymphocyte. Supplementary Fig. 3. Characteristics of TIL products. (A) Expression of CD8, CD4 in CD3 positive cells. (B) Percentage of CD3 + CD56 + NKT, CD3 + CD56 + NK, and CD3 + CD56 − T in CD45 positive cells. (C) Percentage of Tn, TCM, TEM, and TEMRA in T lymphocytes. (D) Expression of CD25, CD28, CD44, CD69, CD137, CD150, and CD 39 in T lymphocytes. (E) Expression of CD 57, PD-1, LAG-3, TIGIT, and TIM-3 in T lymphocytes. (F) Expression of CCR2, CCR5, CCR7, CXCR1, CXCR2, CXCR3 and CX3CR1 in T lymphocytes. LAG-3, Lymphocyte activation gene 3; PD-1, programmed death-1; PDX, patient-derived xenografts; TCM, central memory T cells; TEM, effector memory T cells; TEMRA, CD45RA + effector memory T cells; TIGIT, T cell immunoreceptor with immunoglobulin and ITIM domains; TIL, Tumor-infiltrating lymphocyte; TIM-3, T cell immunoglobulin mucin family member-3; Tn, naïve T. Supplementary Fig. 4. Analysis of peripheral blood pre- and post-infusion of TILs. (A) Summarized analysis of white blood cells, neutrophils and lymphocytes. (B) Summarized analysis of haemoglonbins and platelets. (C) Summarized analysis of IL-6, IL-8, IFN-γ and TNF. (D-L) shows white blood cells, neutrophils, lymphocytes, haemoglonbins, platelets, IL-6, IL-8, IFN-γ, and TNF per patient respectively. TIL, Tumor-infiltrating lymphocyte. Supplementary Fig. 5. The association between clinical response with dynamics of CD8 + /CD4 + ratio. (A) The ORR for the high relative CD8 + /CD4 + ratio group was 50%, and was 0 for the low relative CD8 + /CD4 + ratio group. (B) The DCR for the high relative CD8 + /CD4 + ratio group was 87.5%, and was 50.0% for the low relative CD8 + /CD4 + ratio group.

Additional file 2: Table S1-S2. Table S1-Characteristics of Patients and Administered TILs. Table S2-Best response to treatment.

## Data Availability

Data utilized in this study are immediately available from the corresponding author upon reasonable request.

## Abbreviations

ACT: Adoptive cell therapy
AE: Adverse event
CI: Confidence interval
COVID-19: Coronavirus disease 2019
CR: Complete response
CRS: Cytokine release syndrome
CTCAE: Common Terminology Criteria for Adverse Events
DCR: Disease control rate
DOR: Duration of response
ECOG: Eastern Cooperative Oncology Group
ICI: Immune checkpoint inhibitor
IL: Interleukin
OR: Overall response
ORR: Objective response rate
PD-1: Programmed cell death 1
PD-L1: PD ligand-1
PFS: Progression-free survival
PR: Partial response
RECIST: Response Evaluation Criteria in Solid Tumors
SAE: Serious adverse event
SD: Stable disease
TCM: Central memory T cell
TCR: T cell receptor
TEM: Effector memory T cell
TIL: Tumor-infiltrating lymphocyte
TME: Tumor microenvironment
Treg cells: Regulatory T cells
