# Supplementary figures and images for: IL-2-free tumor-infiltrating lymphocyte therapy with PD-1 blockade demonstrates potent efficacy in advanced gynecologic cancer
Source: BMC Med. 2024 May 20;22:207. doi: 10.1186/s12916-024-03420-0 (PMC11106999; doi:10.1186/s12916-024-03420-0)

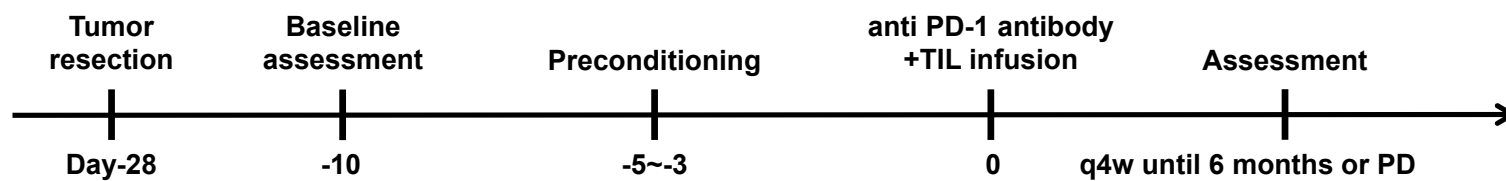

Supplement: Supplementary file 1 — Additional file 1: Figure S1-S5. Fig. S1-Scheme of clinical study. Fig. S2-Characteristics of TIL used in PDX model. Fig. S3-Characteristics of TIL products. Fig. S4-Analysis of peripheral blood pre- and post-infusion of TILs. Fig. S5-The association between clinical response with dynamics of CD8 + /CD4 + ratio. Supplementary Fig. 1. Scheme of clinical study. Day count was relative to TIL infusion. Preconditioning regimen: cyclophosphamide (20 mg/kg/day) from day -5 to day -3, and oral administration of hydroxychloroquine (600 mg once) on day -5. On day 0, following the administration of anti-PD-1 antibody (100 mg, sintilimab, Innovent) patients received a single intravenous TILs. Supplementary Fig. 2. Characteristics of TIL used in PDX model. Flow cytometry analysis of the TIL generating for PDX model. (A) CD45 positive cells in generated cells. (B) Expression of CD3 in CD45 positive cells. (C) analysis of CD45 and CD3 double positive cells. LAG-3, Lymphocyte activation gene 3; PD-1, programmed death-1; PDX, patient-derived xenografts; TCM, central memory T cells; TEM, effector memory T cells; TIL, Tumor-infiltrating lymphocyte. Supplementary Fig. 3. Characteristics of TIL products. (A) Expression of CD8, CD4 in CD3 positive cells. (B) Percentage of CD3 + CD56 + NKT, CD3 + CD56 + NK, and CD3 + CD56 − T in CD45 positive cells. (C) Percentage of Tn, TCM, TEM, and TEMRA in T lymphocytes. (D) Expression of CD25, CD28, CD44, CD69, CD137, CD150, and CD 39 in T lymphocytes. (E) Expression of CD 57, PD-1, LAG-3, TIGIT, and TIM-3 in T lymphocytes. (F) Expression of CCR2, CCR5, CCR7, CXCR1, CXCR2, CXCR3 and CX3CR1 in T lymphocytes. LAG-3, Lymphocyte activation gene 3; PD-1, programmed death-1; PDX, patient-derived xenografts; TCM, central memory T cells; TEM, effector memory T cells; TEMRA, CD45RA + effector memory T cells; TIGIT, T cell immunoreceptor with immunoglobulin and ITIM domains; TIL, Tumor-infiltrating lymphocyte; TIM-3, T cell immunoglobulin mucin family member-3 [file 12916_2024_3420_MOESM1_ESM.zip › Additional file 1 Fig. S1R4.pdf]

**A**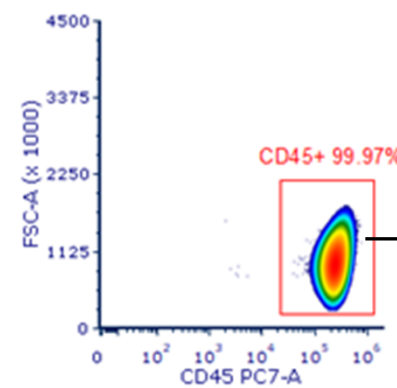**B**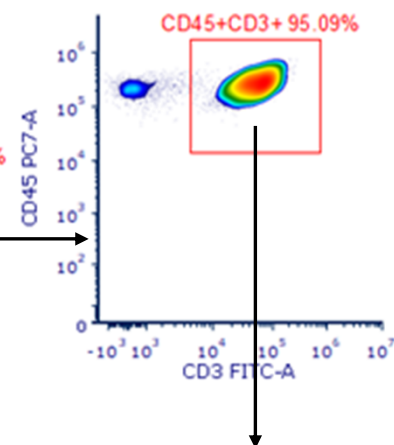**C**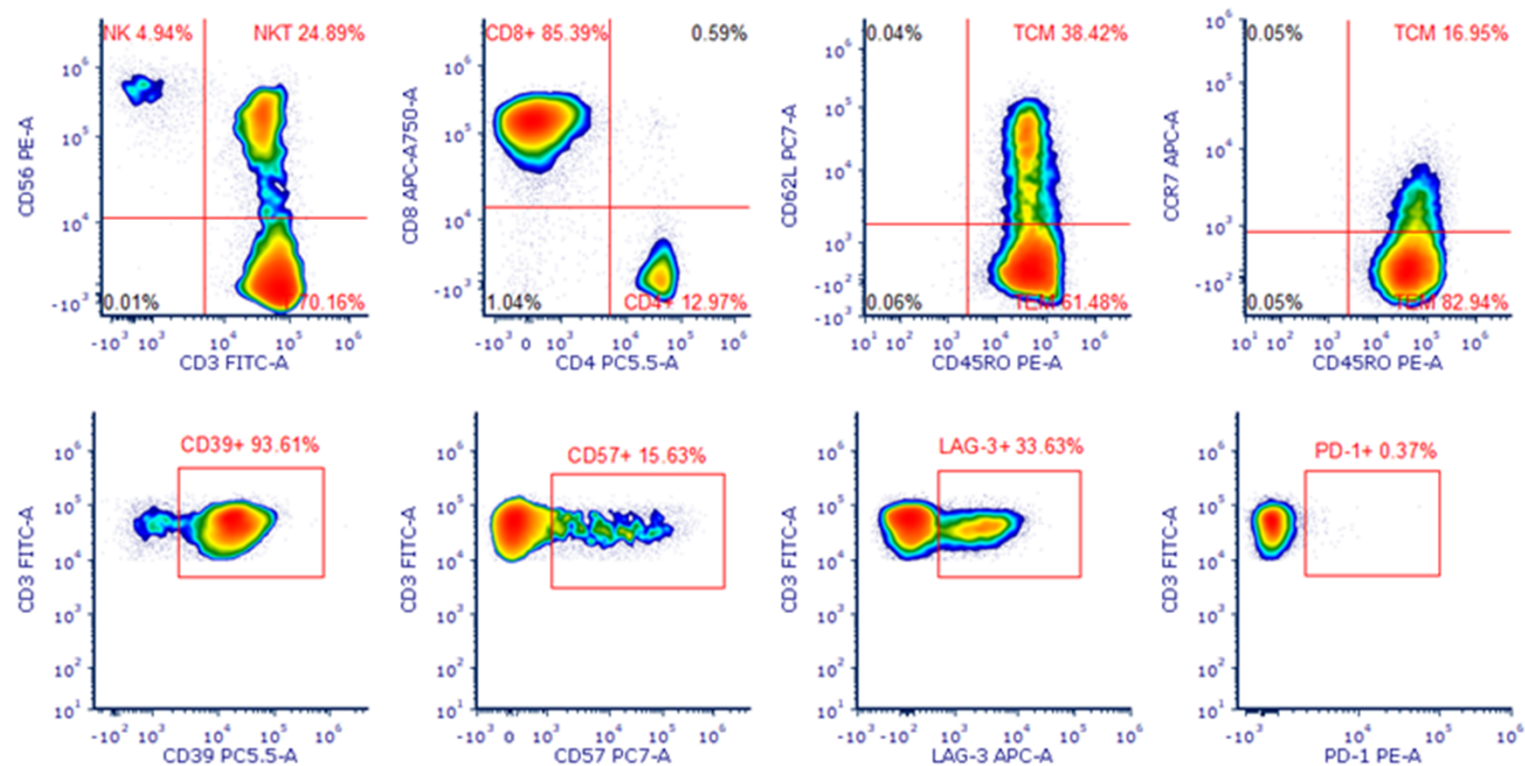

Supplement: Supplementary file 1 — Additional file 1: Figure S1-S5. Fig. S1-Scheme of clinical study. Fig. S2-Characteristics of TIL used in PDX model. Fig. S3-Characteristics of TIL products. Fig. S4-Analysis of peripheral blood pre- and post-infusion of TILs. Fig. S5-The association between clinical response with dynamics of CD8 + /CD4 + ratio. Supplementary Fig. 1. Scheme of clinical study. Day count was relative to TIL infusion. Preconditioning regimen: cyclophosphamide (20 mg/kg/day) from day -5 to day -3, and oral administration of hydroxychloroquine (600 mg once) on day -5. On day 0, following the administration of anti-PD-1 antibody (100 mg, sintilimab, Innovent) patients received a single intravenous TILs. Supplementary Fig. 2. Characteristics of TIL used in PDX model. Flow cytometry analysis of the TIL generating for PDX model. (A) CD45 positive cells in generated cells. (B) Expression of CD3 in CD45 positive cells. (C) analysis of CD45 and CD3 double positive cells. LAG-3, Lymphocyte activation gene 3; PD-1, programmed death-1; PDX, patient-derived xenografts; TCM, central memory T cells; TEM, effector memory T cells; TIL, Tumor-infiltrating lymphocyte. Supplementary Fig. 3. Characteristics of TIL products. (A) Expression of CD8, CD4 in CD3 positive cells. (B) Percentage of CD3 + CD56 + NKT, CD3 + CD56 + NK, and CD3 + CD56 − T in CD45 positive cells. (C) Percentage of Tn, TCM, TEM, and TEMRA in T lymphocytes. (D) Expression of CD25, CD28, CD44, CD69, CD137, CD150, and CD 39 in T lymphocytes. (E) Expression of CD 57, PD-1, LAG-3, TIGIT, and TIM-3 in T lymphocytes. (F) Expression of CCR2, CCR5, CCR7, CXCR1, CXCR2, CXCR3 and CX3CR1 in T lymphocytes. LAG-3, Lymphocyte activation gene 3; PD-1, programmed death-1; PDX, patient-derived xenografts; TCM, central memory T cells; TEM, effector memory T cells; TEMRA, CD45RA + effector memory T cells; TIGIT, T cell immunoreceptor with immunoglobulin and ITIM domains; TIL, Tumor-infiltrating lymphocyte; TIM-3, T cell immunoglobulin mucin family member-3 [file 12916_2024_3420_MOESM1_ESM.zip › Additional file 1 Fig. S2R4.pdf]

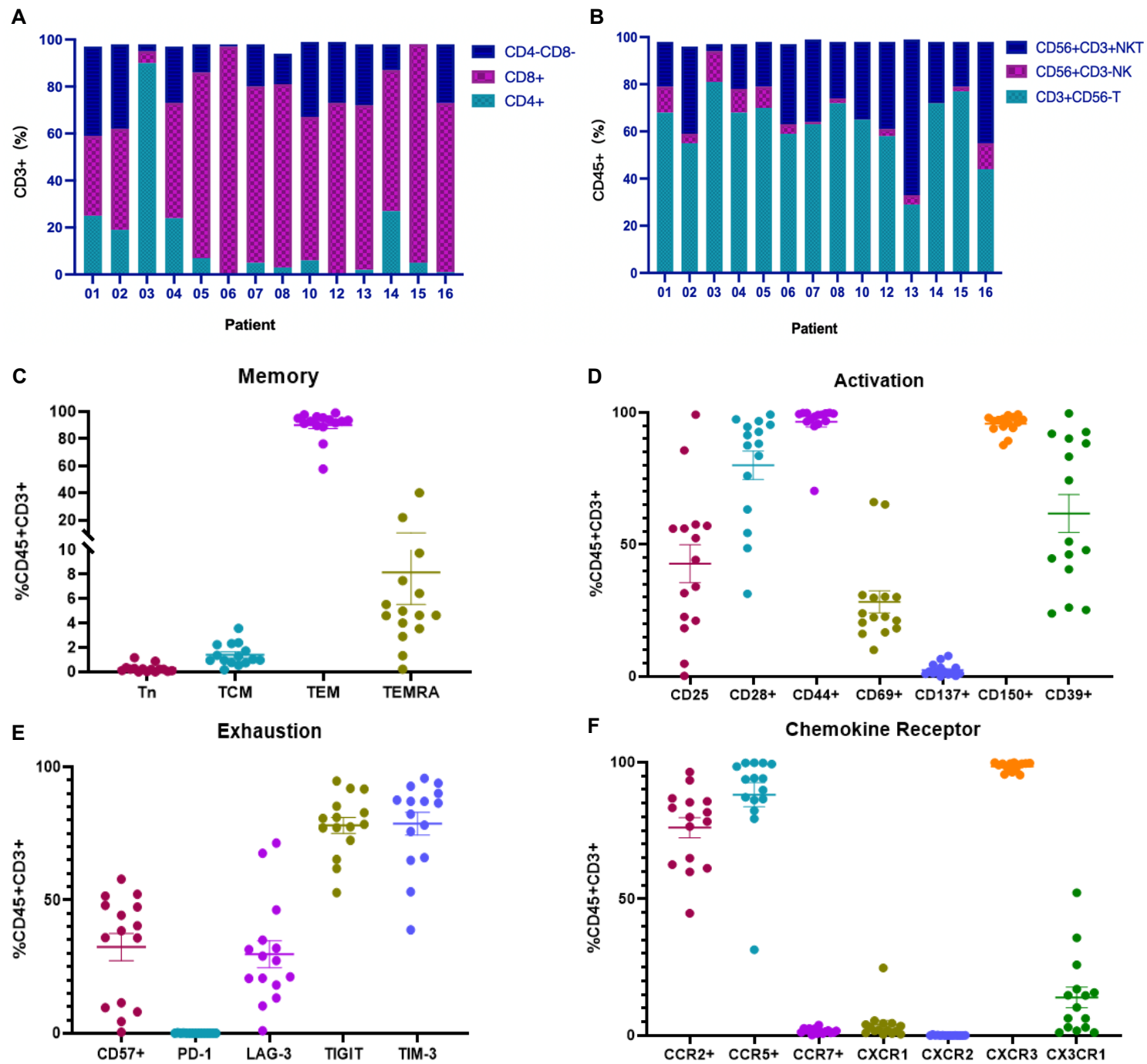

Supplement: Supplementary file 1 — Additional file 1: Figure S1-S5. Fig. S1-Scheme of clinical study. Fig. S2-Characteristics of TIL used in PDX model. Fig. S3-Characteristics of TIL products. Fig. S4-Analysis of peripheral blood pre- and post-infusion of TILs. Fig. S5-The association between clinical response with dynamics of CD8 + /CD4 + ratio. Supplementary Fig. 1. Scheme of clinical study. Day count was relative to TIL infusion. Preconditioning regimen: cyclophosphamide (20 mg/kg/day) from day -5 to day -3, and oral administration of hydroxychloroquine (600 mg once) on day -5. On day 0, following the administration of anti-PD-1 antibody (100 mg, sintilimab, Innovent) patients received a single intravenous TILs. Supplementary Fig. 2. Characteristics of TIL used in PDX model. Flow cytometry analysis of the TIL generating for PDX model. (A) CD45 positive cells in generated cells. (B) Expression of CD3 in CD45 positive cells. (C) analysis of CD45 and CD3 double positive cells. LAG-3, Lymphocyte activation gene 3; PD-1, programmed death-1; PDX, patient-derived xenografts; TCM, central memory T cells; TEM, effector memory T cells; TIL, Tumor-infiltrating lymphocyte. Supplementary Fig. 3. Characteristics of TIL products. (A) Expression of CD8, CD4 in CD3 positive cells. (B) Percentage of CD3 + CD56 + NKT, CD3 + CD56 + NK, and CD3 + CD56 − T in CD45 positive cells. (C) Percentage of Tn, TCM, TEM, and TEMRA in T lymphocytes. (D) Expression of CD25, CD28, CD44, CD69, CD137, CD150, and CD 39 in T lymphocytes. (E) Expression of CD 57, PD-1, LAG-3, TIGIT, and TIM-3 in T lymphocytes. (F) Expression of CCR2, CCR5, CCR7, CXCR1, CXCR2, CXCR3 and CX3CR1 in T lymphocytes. LAG-3, Lymphocyte activation gene 3; PD-1, programmed death-1; PDX, patient-derived xenografts; TCM, central memory T cells; TEM, effector memory T cells; TEMRA, CD45RA + effector memory T cells; TIGIT, T cell immunoreceptor with immunoglobulin and ITIM domains; TIL, Tumor-infiltrating lymphocyte; TIM-3, T cell immunoglobulin mucin family member-3 [file 12916_2024_3420_MOESM1_ESM.zip › Additional file 1 Fig. S3R4.pdf]

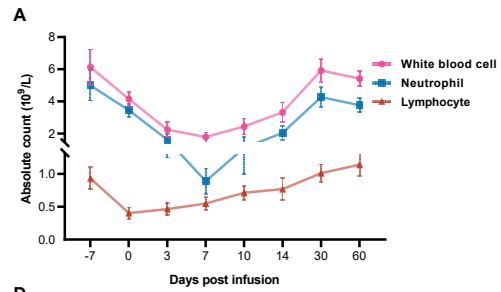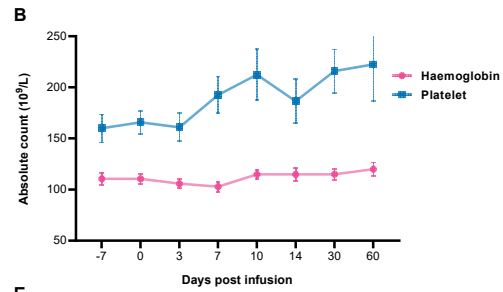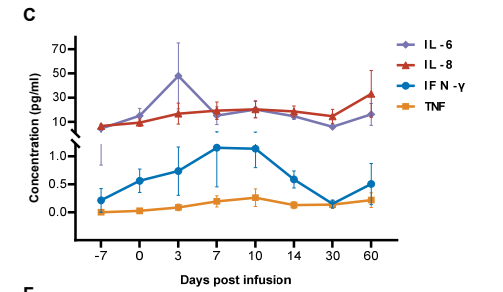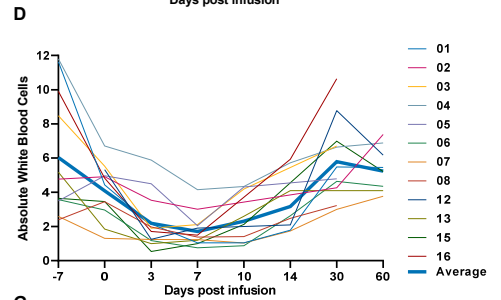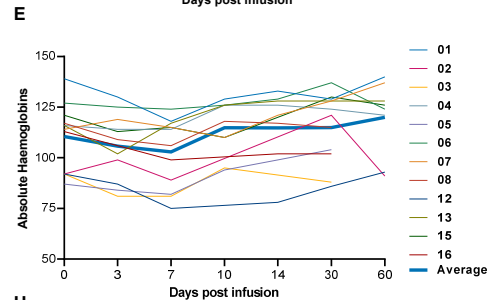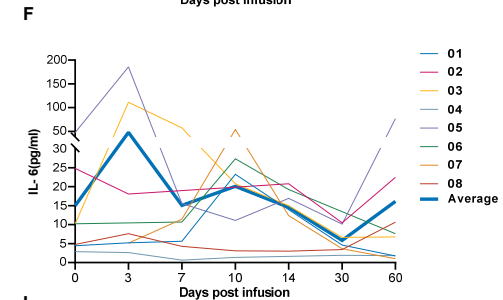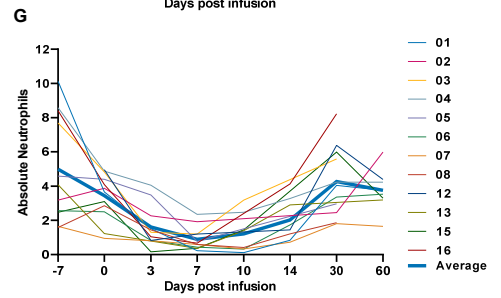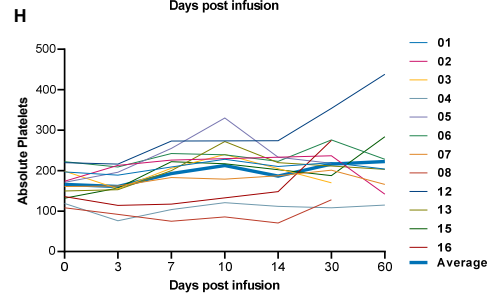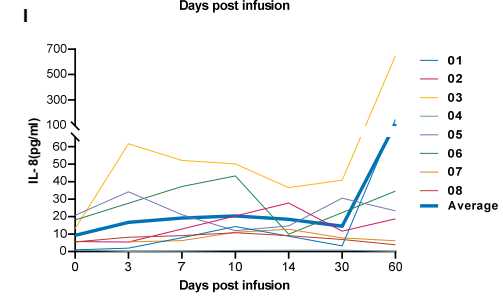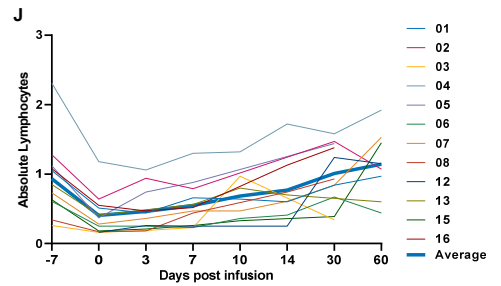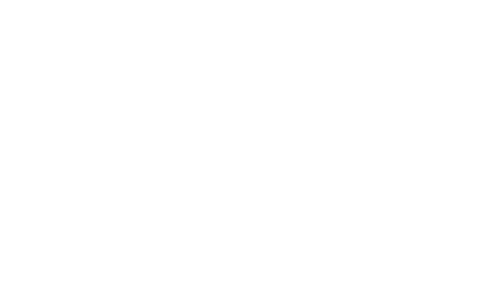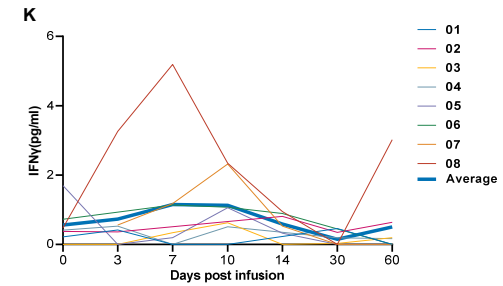

Supplement: Supplementary file 1 — Additional file 1: Figure S1-S5. Fig. S1-Scheme of clinical study. Fig. S2-Characteristics of TIL used in PDX model. Fig. S3-Characteristics of TIL products. Fig. S4-Analysis of peripheral blood pre- and post-infusion of TILs. Fig. S5-The association between clinical response with dynamics of CD8 + /CD4 + ratio. Supplementary Fig. 1. Scheme of clinical study. Day count was relative to TIL infusion. Preconditioning regimen: cyclophosphamide (20 mg/kg/day) from day -5 to day -3, and oral administration of hydroxychloroquine (600 mg once) on day -5. On day 0, following the administration of anti-PD-1 antibody (100 mg, sintilimab, Innovent) patients received a single intravenous TILs. Supplementary Fig. 2. Characteristics of TIL used in PDX model. Flow cytometry analysis of the TIL generating for PDX model. (A) CD45 positive cells in generated cells. (B) Expression of CD3 in CD45 positive cells. (C) analysis of CD45 and CD3 double positive cells. LAG-3, Lymphocyte activation gene 3; PD-1, programmed death-1; PDX, patient-derived xenografts; TCM, central memory T cells; TEM, effector memory T cells; TIL, Tumor-infiltrating lymphocyte. Supplementary Fig. 3. Characteristics of TIL products. (A) Expression of CD8, CD4 in CD3 positive cells. (B) Percentage of CD3 + CD56 + NKT, CD3 + CD56 + NK, and CD3 + CD56 − T in CD45 positive cells. (C) Percentage of Tn, TCM, TEM, and TEMRA in T lymphocytes. (D) Expression of CD25, CD28, CD44, CD69, CD137, CD150, and CD 39 in T lymphocytes. (E) Expression of CD 57, PD-1, LAG-3, TIGIT, and TIM-3 in T lymphocytes. (F) Expression of CCR2, CCR5, CCR7, CXCR1, CXCR2, CXCR3 and CX3CR1 in T lymphocytes. LAG-3, Lymphocyte activation gene 3; PD-1, programmed death-1; PDX, patient-derived xenografts; TCM, central memory T cells; TEM, effector memory T cells; TEMRA, CD45RA + effector memory T cells; TIGIT, T cell immunoreceptor with immunoglobulin and ITIM domains; TIL, Tumor-infiltrating lymphocyte; TIM-3, T cell immunoglobulin mucin family member-3 [file 12916_2024_3420_MOESM1_ESM.zip › Additional file 1 Fig. S4R4.pdf]

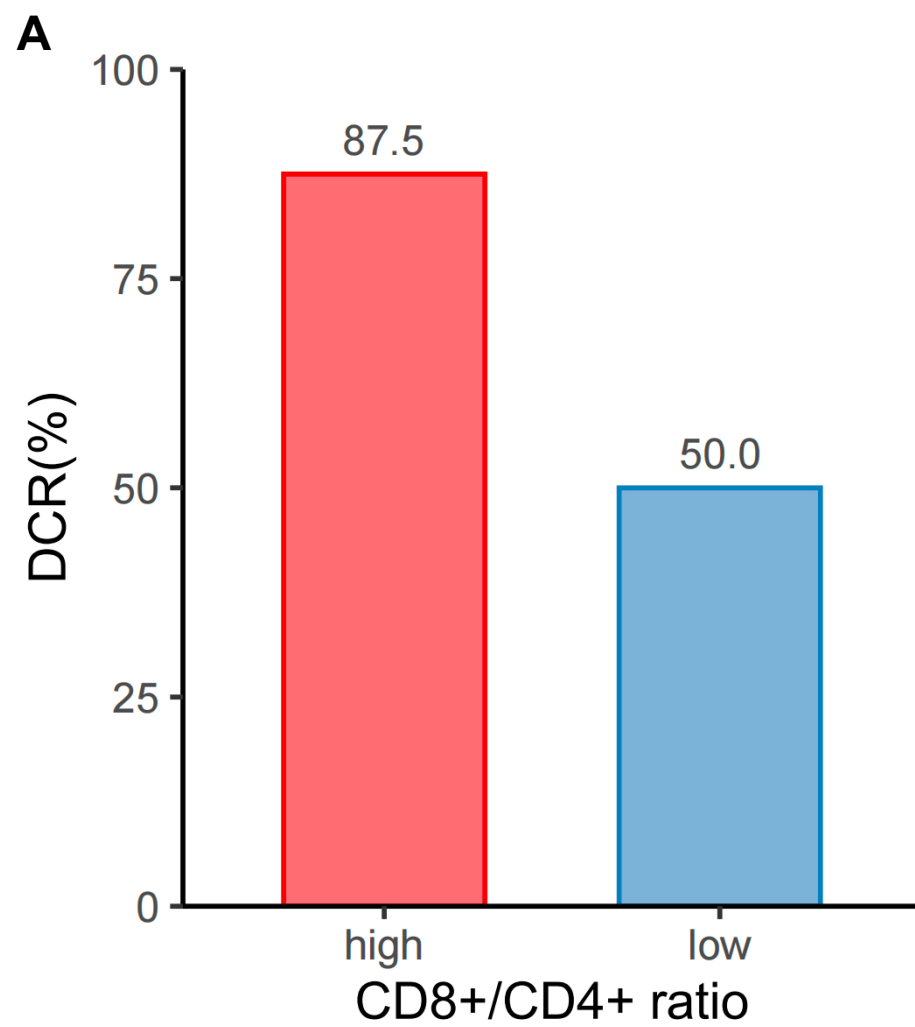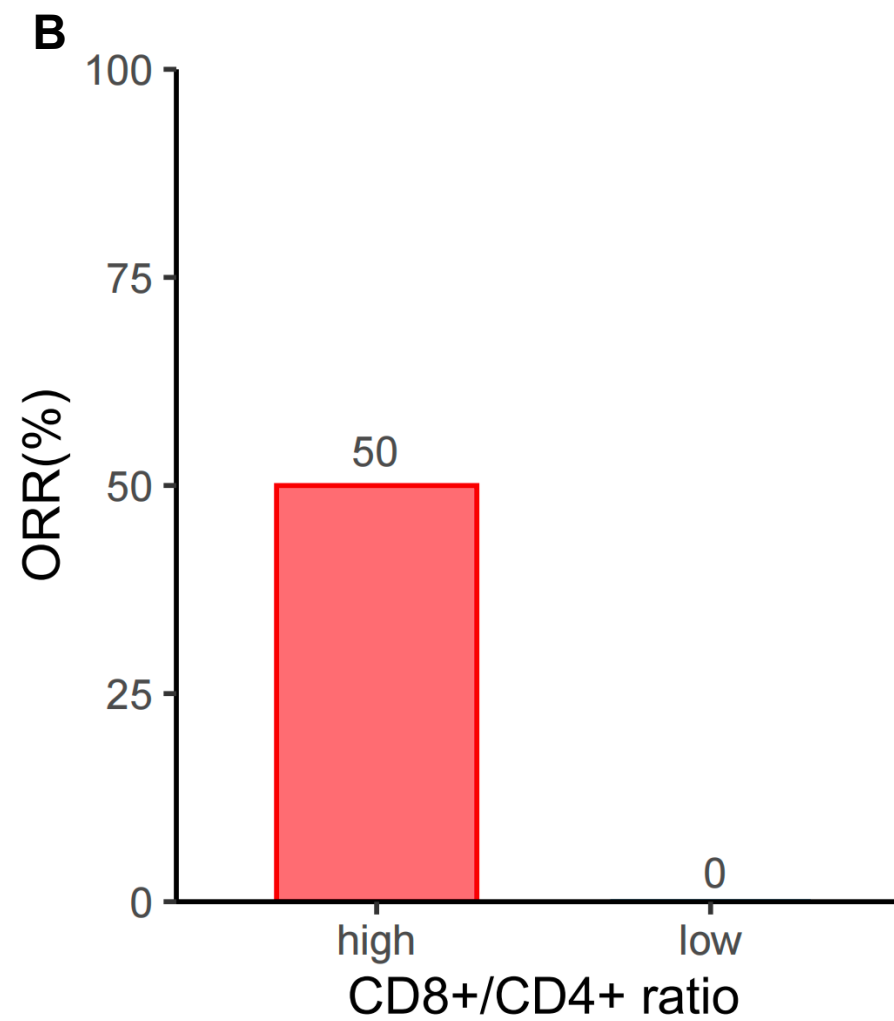

Supplement: Supplementary file 1 — Additional file 1: Figure S1-S5. Fig. S1-Scheme of clinical study. Fig. S2-Characteristics of TIL used in PDX model. Fig. S3-Characteristics of TIL products. Fig. S4-Analysis of peripheral blood pre- and post-infusion of TILs. Fig. S5-The association between clinical response with dynamics of CD8 + /CD4 + ratio. Supplementary Fig. 1. Scheme of clinical study. Day count was relative to TIL infusion. Preconditioning regimen: cyclophosphamide (20 mg/kg/day) from day -5 to day -3, and oral administration of hydroxychloroquine (600 mg once) on day -5. On day 0, following the administration of anti-PD-1 antibody (100 mg, sintilimab, Innovent) patients received a single intravenous TILs. Supplementary Fig. 2. Characteristics of TIL used in PDX model. Flow cytometry analysis of the TIL generating for PDX model. (A) CD45 positive cells in generated cells. (B) Expression of CD3 in CD45 positive cells. (C) analysis of CD45 and CD3 double positive cells. LAG-3, Lymphocyte activation gene 3; PD-1, programmed death-1; PDX, patient-derived xenografts; TCM, central memory T cells; TEM, effector memory T cells; TIL, Tumor-infiltrating lymphocyte. Supplementary Fig. 3. Characteristics of TIL products. (A) Expression of CD8, CD4 in CD3 positive cells. (B) Percentage of CD3 + CD56 + NKT, CD3 + CD56 + NK, and CD3 + CD56 − T in CD45 positive cells. (C) Percentage of Tn, TCM, TEM, and TEMRA in T lymphocytes. (D) Expression of CD25, CD28, CD44, CD69, CD137, CD150, and CD 39 in T lymphocytes. (E) Expression of CD 57, PD-1, LAG-3, TIGIT, and TIM-3 in T lymphocytes. (F) Expression of CCR2, CCR5, CCR7, CXCR1, CXCR2, CXCR3 and CX3CR1 in T lymphocytes. LAG-3, Lymphocyte activation gene 3; PD-1, programmed death-1; PDX, patient-derived xenografts; TCM, central memory T cells; TEM, effector memory T cells; TEMRA, CD45RA + effector memory T cells; TIGIT, T cell immunoreceptor with immunoglobulin and ITIM domains; TIL, Tumor-infiltrating lymphocyte; TIM-3, T cell immunoglobulin mucin family member-3 [file 12916_2024_3420_MOESM1_ESM.zip › Additional file 1 Fig. S5R4.pdf]
