# Supplementary material for: IL-2-free tumor-infiltrating lymphocyte therapy with PD-1 blockade demonstrates potent efficacy in advanced gynecologic cancer
Source: BMC Med. 2024 May 20;22:207. doi: 10.1186/s12916-024-03420-0 (PMC11106999; doi:10.1186/s12916-024-03420-0)
Supplement: Supplementary file 2 — Additional file 2: Table S1-S2. Table S1-Characteristics of Patients and Administered TILs. Table S2-Best response to treatment. [file 12916_2024_3420_MOESM2_ESM.zip › Additional file 2 Table S1R4.docx]

**Additional file 6: Table S1. Characteristics of Patients and Administered TILs**

| **Patient** | **Age** | **Baseline ECOG score** | **Type of cancer** | **Histology** | **Sites of Disease** | **Sum diameter of target lesion (mm)** | **Prior Systemic Treatment** | **Cells (×10^9^)** | **CD8^+^/CD3^+^ (%)** | **Best response** | **Duration (month)** |
| --- | --- | --- | --- | --- | --- | --- | --- | --- | --- | --- | --- |
| **1** | 52 | 1 | CC | CESC | Bladder, iliac lymph nodes | 17 | Paclitaxel, cisplatin, bevacizumab. | 18.8 | 72 | CR | 19.5 |
| **2** | 51 | 3 | OC | MOC | Sigmoid colon, rectum, omentum and peritoneum | 22 | Cisplatin, carboplatin. | 14.9 | 60 | SD | 3.2 |
| **3** | 58 | 3 | OC | HGSOC | Vaginal cuff | 65 | Paclitaxel, cisplatin, olaparib, bevacizumab, doxorubicin, niraparib, camrelizumab. | 12.5 | 43 | SD | 5.3 |
| **4** | 37 | 1 | CC | CESC | Bladder, iliac lymph nodes | 36 | Paclitaxel, cisplatin. | 24 | 93 | PR | 16.5 |
| **5** | 42 | 2 | CC | CESC | Retroperitoneal, omentum, abdominal cavity, liver, lung, iliac lymph nodes | 87 | Paclitaxel, cisplatin, carboplatin, pemetrexed, cadonilimab, apatinib mesylate, anlotinib. | 50.5 | 97 | SD | 6.3 |
| **6** | 40 | 2 | CC | CESC | Peritoneal, retroperitoneal | 10 | Docetaxel, carboplatin, paclitaxel, nedaplatin, sintilimab, bevacizumab, eribulin. | 13.4 | 97 | SD | 5.3 |
| **7** | 38 | 1 | EC | EC | Retroperitoneal, liver, pelvic cavity, abdominal cavity | 24 | Carboplatin, paclitaxel, cisplatin. | 26.4 | 5 | CR | 15.4 |
| **8** | 58 | 1 | OC | HGSOC | Basin of abdominal cavity; retroperitoneal, bilateral iliac vessels adjacent to the inguinal | 33 | Paclitaxel, cisplatin, bevacizumab, liposomal doxorubicin, anastrozole, nivolumab, olaparib, cyclophosphamide. | 27.5 | 79 | PR | 13.7 |
| **10** | 33 | 1 | OC | EEOC | Pelvic cavity, peritoneum, pelvic wall | 48 | Paclitaxel, cisplatin, bevacizumab, niraparib. | 15.4 | 70 | SD | 12.5 |
| **12** | 51 | 2 | OC | OC | Vaginal cuff, pelvic cavity, pelvic wall, liver, abdominal cavity, spleno-gastric junction, retroperitoneal | 79 | Paclitaxel, lobaplatin, olaparib, bevacizumab, doxorubicin, gemcitabine, cisplatin. | 23 | 73 | PD | 10.6 |
| **13** | 56 | 2 | CC | CESC | Liver, lung, pleura | 63 | Sintilimab. | 38.5 | 61 | SD | 8.5 |
| **14** | 65 | 2 | OC | OC | Abdominal wall, iliac lymph nodes, peritoneal, omentum, retroperitoneal | 84 | Paclitaxel, cisplatin, carboplatin, bevacizumab, olaparib, gemcitabine, etoposide. | 15.3 | 78 | PD | 2.8 |
| **15** | 65 | 2 | EC | EC | Anterior sacral fascia | 23 | Paclitaxel, carboplatin, gemcitabine, carboplatin, pembrolizumab. | 13.8 | 19 | CR | 5.2 |
| **16** | 63 | 3 | OC | OC | Peritoneal, omentum, pelvic cavity, retroperitoneal, subphrenic | 20 | Paclitaxel, cisplatin, bevacizumab, gemcitabine, carboplatin, epirubicin, bevacizumab, docetaxel. | 26.5 | 73 | PD | 2.5 |
