# Supplementary material for: IL-2-free tumor-infiltrating lymphocyte therapy with PD-1 blockade demonstrates potent efficacy in advanced gynecologic cancer
Source: BMC Med. 2024 May 20;22:207. doi: 10.1186/s12916-024-03420-0 (PMC11106999; doi:10.1186/s12916-024-03420-0)
Supplement: Supplementary file 2 — Additional file 2: Table S1-S2. Table S1-Characteristics of Patients and Administered TILs. Table S2-Best response to treatment. [file 12916_2024_3420_MOESM2_ESM.zip › Additional file 2 Table S2R4.docx]

**Supplementary Table 2. Best response to treatment**

| Variable | **Investigator assessment (n=14)** | |
| --- | --- | --- |
|  | **n** | **Percentage of patients** |
| Best response |  |  |
| Complete response | 3 | 21% |
| Partial response | 2 | 14% |
| Stable disease | 5 | 36% |
| Disease progression | 4 | 29% |
| Overall response | 5 | 36% (16.3-61.2) |
| Disease control | 10 | 71% (45.4-88.3) |

Percentages are % or % (95% CI).
